# Systematic molecular analysis of the human secretome and membrane proteome in gastrointestinal adenocarcinomas

**DOI:** 10.1111/jcmm.17338

**Published:** 2022-04-29

**Authors:** Adeel ur Rehman, Per Olof Olsson, Aleena Akhtar, Arshad Ahmed Padhiar, Hanyang Liu, Yi Dai, Yu Gong, Yan Zhou, Naveed Khan, Haojun Yang, Liming Tang

**Affiliations:** ^1^ Department of General Surgery Changzhou No. 2 People's Hospital affiliated with Nanjing Medical University Changzhou China; ^2^ UAE Biotech Research Center Abu Dhabi United Arab Emirates; ^3^ 12412 Changzhou University Changzhou China; ^4^ 7712 Department of Ecology and Evolutionary Biology University of Connecticut Storrs Connecticut USA; ^5^ Charité‑University Medical Center Department of Hematology, Oncology and Tumor Immunology Virchow Campus, and Molecular Cancer Research Center Berlin Germany; ^6^ CAS‐MPG Partner Institute for Computational Biology Shanghai Institute of Biological Sciences University of Chinese Academy of Science Chinese Academy of Science Shanghai China

**Keywords:** cancer secretome, gastric cancer, IL1RAP, membrane protein, RNA sequencing, secretory protein

## Abstract

The human secretome and membrane proteome are a large source of cancer biomarkers. Membrane‐bound and secreted proteins are promising targets for many clinically approved drugs, including for the treatment of tumours. Here, we report a deep systematic analysis of 957 adenocarcinomas of the oesophagus, stomach, colon and rectum to examine the cancer‐associated human secretome and membrane proteome of gastrointestinal tract adenocarcinomas (GIACs). Transcriptomic data from these GIACs were applied to an innovative majority decision‐based algorithm. We quantified significantly expressed protein‐coding genes. Interestingly, we found a consistent pattern in a small group of genes found to be overexpressed in GIACs, which were associated with a cytokine–cytokine interaction pathway (CCRI) in all four cancer subtypes. These CCRI associated genes, which spanned both one secretory and one membrane isoform were further analysed, revealing a putative biomarker, interleukin‐1 receptor accessory protein (IL1RAP), which indicated a poor overall survival, a positive correlation with cancer stemness and a negative correlation with several kinds of T cells. These results were further validated in vitro through the knockdown of IL1RAP in two human gastric carcinoma cell lines, which resulted in a reduced indication of cellular proliferation, migration and markers of invasiveness. Following IL1RAP silencing, RNA seq results showed a consistent pattern of inhibition related to CCRI, proliferation pathways and low infiltration of regulatory T cells (Tregs) and CD8 naive cells. The significance of the human secretome and membrane proteome is elucidated by these findings, which indicate IL1RAP as a potential candidate biomarker for cytokine‐mediated cancer immunotherapy in gastric carcinoma.

## 1 INTRODUCTION

Cancer patient survival is positively correlated with early diagnosis.^1^ Next‐generation sequencing and other sophisticated tools have improved the sensitivity and accuracy of the detection of cancer biomarkers for diagnostic use.^2^ Recently biological fluids, for example urine, blood and cerebrospinal fluids have garnered more attention as a source for biomarker discovery, due to the less‐invasive nature and relative ease of testing, when compared to tissue biopsies.^3^, ^4^ A portion of the human proteome consists of secreted proteins, these proteins are found in the extracellular matrix and biological fluids. Identification and selection of protein biomarkers from the cellular secretome, with subsequent verification is a promising approach due to the relative ease of access and analysis.^5^, ^6^

A small portion of the human proteome contains membrane proteins with aberrant expression linked with tumour progression and metastasis.^7^ Enzymes, ion channels, transport and adhesion molecules are included in the examples of these membrane proteins.^8^ Secretory and membrane proteins are both involved in a variety of biochemical and physiological regulatory pathways. Having primarily been profiled using proteomic approaches, the cancer secretome and membrane proteome publications are largely comprised of the investigation of interstitial fluid from tumours or radially accessible fluids, for example saliva, urine, plasma or blood or *in vitro* cell line‐based studies. The membrane proteome and secretome are thought to be a rich source of potential biomarkers for malignant growth and different diseases. Numerous studies have been performed to examine these protein subtypes in the search for putative cancer biomarkers. Welsh et al. (2003)^9^ compared microarray gene expression data from 150 tumours spanning 10 tissues of origin to those of 46 normal tissue samples using the gene ontology (GO) terminology correlated with extracellular position and protein sequence patterns. Biomarker candidates have previously been investigated, which measure high gene or protein expression levels in tumour samples or serum of cancer patients. Other bioinformatics‐based studies including prostate, lung, pancreatic, colon and ovarian cancers have been used to predict secreted cancer biomarker candidates.^10^, ^11^ Several publications have illustrated the accuracy of employing a bioinformatics‐based method to predict and identify proteomic biofluid targets and subsequently develop promising cancer‐specific candidate biomarkers. These studies have been limited to a small number of samples, cell lines and/or cancer types; usually based on outdated microarray data rather than RNA sequencing data, and little or no research into the biological functions associated with the proposed biomarkers have been performed. The criteria for biomarkers identification have been well defined in clinical terms and require substantial clinical and analytic validation before being used.^12^

Cytokines (interleukins), growth factors, coagulation factors and hormones are among the most commonly known secreted proteins.^13^ The identification, characterization and quantification of secretory and membrane proteins (SMPs) in cancer is an emerging field of onco‐proteomics.^14^ Cell–cell adhesion and signalling are two elements in the hallmarks of cancer which are reliant on SMPs.^15^, ^16^ SMPs have been implicated in tumour invasion, metastasis and tumorigenicity in many studies,^17^, ^18^, ^19^, ^20^, ^21^ and have been identified as attractive diagnostic and therapeutic markers for a wide range of tumours.^5^, ^8^, ^22^ Many publications have been focused on SMPs and the production of massive amounts of data in a quest for cancer biomarkers.^23^ Although there are presently no detailed studies of SMPs in tumours, the research into cancer secretory and membrane proteins offers significant promise for the development of cancer detection and therapy‐related biomarkers.^12^

Interleukin‐1 receptor accessory protein (IL1RAP) spanning both secretory and membrane protein isoforms and co‐receptor of the interleukin (IL) IL1 and IL33 receptors involved in IL1 signalling, activates various signalling systems involved in proliferation and inflammation. IL1RAP has been shown to be overexpressed in various cancer subtypes and even suggested to play a role in disease relapse.^24^ IL1RAP is a novel target for antibody therapy, which suppresses tumour cell proliferation in vitro and in vivo.^25^, ^26^ Overall, IL1RAP appears to be a key membrane‐bound tumour antigen for targeting antibody‐mediated selective treatment. The identification and potential preclinical significance of secretory and membrane proteins, including the novel function and mechanistic roles of IL1RAP in the context of gastric cancer pathogenesis are described here.

## 2 METHODS

### 2.1 RNA sequencing data

The UCSC cancer genome browser^27^ was used to download gene expression TCGA ESCA (oesophageal carcinoma), TCGA STAD (stomach adenocarcinoma), TCGA COAD (colon adenocarcinoma), READ (rectum adenocarcinoma) and GTEx (the genotype‐tissue expression project) data. The TCGA collected fresh and frozen samples from 957 primary tumours (182 ESCA, 408 STAD, 275 COAD and 92 READ) GIACs. In addition, there were 100 normal adjacent tissues data collected. All patients gave their informed consent, and local institutional review boards approved the collections. Samples that had no detectable expression levels were eliminated for further analysis.

A majority decision‐based algorithm^13^ was used to identify secretory and membrane proteins from GIACs. In total, seven distinct algorithms may be used to predict human membrane proteins. The algorithms used to make these predictions included: THUMBUP, MEMSAT3 (MEMSAT‐SVM), GPCRHMM, TMHMM, SPOCTOPUS, SCAMPI and Phobius. A membrane protein is defined as any protein that has at least one transmembrane region with overlapping predictions from four of the seven algorithms. SPOCTOPUS, Phobius and SignalP4.0 were used to predict secretory proteins. A secretory protein was defined as any protein that included at least one signal peptide with overlapping predictions from two of the three algorithms.

### 2.2 Enrichment analysis

The DAVID v6.8 (the database for annotation, visualization and integrated discovery) was used to upload each list of the secretory, membrane and secretory membrane isoforms and analysis was conducted using default parameters for Homo sapiens.^28^

### 2.3 Enrichment and pathway analysis

To decode functionally categorized and pathway annotation networks, the ClueGO v2.5.7^29^ Cytoscape v3.8.2 program was utilized. The statistical significance of the enrichment term is shown by the node text size, which is based on the immune system process‐EBI‐UniProt and KEGG^30^ data sets and the computed *p* values using Fisher's exact test. The groups were defined using a kappa value of 0.4. Nodes with the same colour show connections to distinct groupings.

### 2.4 Survival analysis

The GEPIA (V2) online tool^31^ was used to investigate the relationship between gene expression levels and the survival of patients from which gastric cancer samples were obtained. The Mantel–cox test and the hazard ratio with 95 percent confidence intervals were used to compare cancer patient cohorts, and log‐rank *p* values <0.05 were considered significant.

### 2.5 Cell lines and cell culture

The human gastric cancer cell lines BGC823 and HGC‐27, obtained from the Cell Resource Center of Shanghai Institute of Biochemistry and Cell Biology and maintained in Dulbecco's modified Eagle's medium (Gibco™) supplemented with 50 µg/ml streptomycin, 50 IU penicillin and 10% FBS (HyClone).

### 2.6 Transfection

All siRNA and their respective negative control were purchased from GenePharma. BGC‐823 and HGC‐27 cells were plated at the same densities 12 h before transfection. Cells were transfected with Negative Control siRNA or IL1RAP‐siRNA (pooled) using Lipofectamine 3000 (Thermo Fisher) according to the manufacturer's instructions.

### 2.7 Gene expression analysis

RNA was isolated using RNA‐simple total RNA kit (TIANGEN) and converted into cDNA using PrimeScript RT Reagent Kit (TAKARA). Real‐time PCR reactions were performed using AceQ^®^ qPCR SYBR^®^ Green Master Mix (Vazyme) and on a ViiA 7 Real‐time PCR system (Applied Biosystems). Primer sequences are available upon request. Relative expression levels were calculated for each gene using the Ct method.

### 2.8 Cell migration assay

Cell migration analysis was performed in BGC‐823 and HGC‐27 cells after 72‐h siRNA transfection. A scratch, simulating a wound was created by cutting the cell monolayer longitudinally with a 10‐μl pipette tip. Cells were allowed to migrate into the ‘wound’ for indicated time points. Image analysis to calculate wound closure was performed using ImageJ (Fiji). Images were acquired using a 10× microscope objective at indicated time points for 36 h.

### 2.9 Cell proliferation assay

The cell proliferation of BGC‐823 and HGC‐27 cells was tested by using the cell proliferation assay (Dojindo). The cells were seeded on a 96‐well plate at a density of 2000 cells per well. Subsequently, 10 µl of CCK8 solution were added into wells that contained 100 µl growth medium. Cells were incubated at 37°C with 5% CO2 for 4 h. Absorbance values at 450 nm were measured using a spectrophotometer (Epoch).

### 2.10 Transwell migration assay

Transwell chambers (8 µm; polycarbonate membrane) were purchased from Corning, Costar. BGC‐823 and HGC‐27 cells were seeded in the upper chambers and cultured with the serum‐free medium. Complete DMEM medium containing 10% serum was added to the lower chamber. Cells in the upper chamber were removed after 24 h of culture, and invasive cells embedded in the transwell membrane were preserved with 4% paraformaldehyde, stained with crystal violet for 5 min and examined under a microscope. The data were analysed using ImageJ software. The data are presented as means SEM.

### 2.11 RNA sequencing

Total RNA was extracted from cells (BCG‐823 and HGC‐27) using MagZol (AnGen Biotech), according to the manufacturer's instructions. The quantity and integrity of RNA yield were evaluated independently using the Agilent 2200 TapeStation (Agilent Technologies). Enrichment of mRNA was done using oligodT and fragmented to 200 bp following NEBNext^®^ Poly(A) mRNA Magnetic Isolation Module (NEB) instructions. Synthesis of cDNA and adaptor ligation and subsequent enrichment was performed using the NEBNext^®^ Ultra™ RNA Library Prep Kit for Illumina, as instructed. Agilent 2200 TapeStation and Qubit (Thermo Fisher Scientific) were used to analyse the purified library products. The material was sequenced on the Illumina (Illumina) with 150 bp read length in paired‐end mode. Library preparation and sequencing were performed.

After removing the adapter, ploy‐N and low‐quality readings from the raw data, clean reads were obtained. With default settings, HISAT2 was used to align the clean reads to the human reference genome hg19.^32^ HTSeq v0.12.4 was used to turn aligned short reads into the read counts for each gene. DEseq/DESeq2/edgeR/DEGseq software was then used to identify statistically significant differentially expressed genes using an adjusted (|log2(FoldChange)|>1 and *q*‐value <0.05). Finally, for the differentially expressed genes in various groups, a hierarchical clustering analysis was done using the R language package gplots. Gene ontology (GO) and Kyoto Encyclopaedia of Genes (KEGG) pathway enrichment analyses were performed using the ‘clusterProfiler’ package in R Bioconductor or Gene Set Enrichment Analysis (GSEA) software to identify the gene ontology (GO) annotations and enrichment pathways in differentially expressed genes.^33^, ^34^, ^35^ Transcription factor motif enrichment and lncRNA sets were analysed using g:profiler and LnSEA database.^36^, ^37^, ^38^ To estimate the immune infiltration status among RNA seq results and TCGA data, we utilized CIBERSORT, TIMER and TIP.^39^, ^40^, ^41^

## 3 RESULTS

### 3.1 Gastric cancer‐associated human secretome and membrane proteome

**FIGURE 1:**
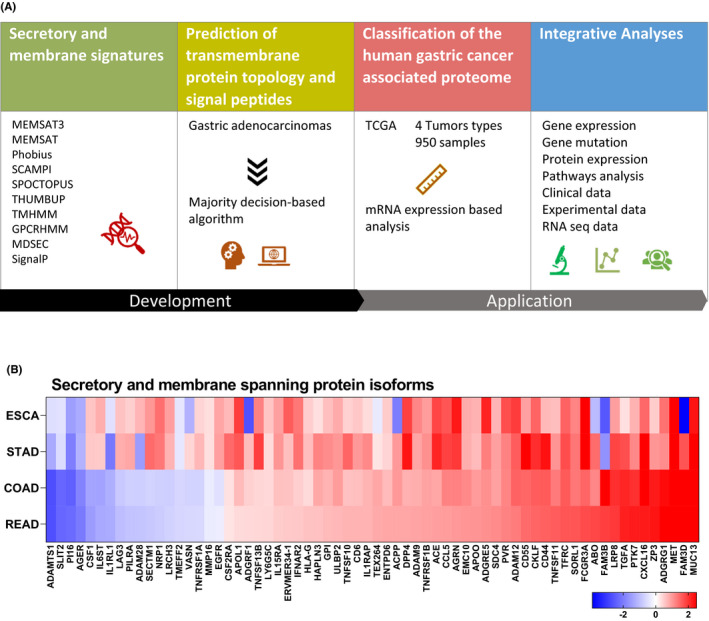
Identification of membrane and secretory proteins from gastrointestinal adenocarcinomas. (A) Overall strategy. Secretory and membrane proteins (SMPs) from each GIACs were compared with known oncology and clinical data. (B) The differential expression of protein‐coding genes with both secretory and membrane isoforms (*q*‐value ≤0.05)

**FIGURE 2:**
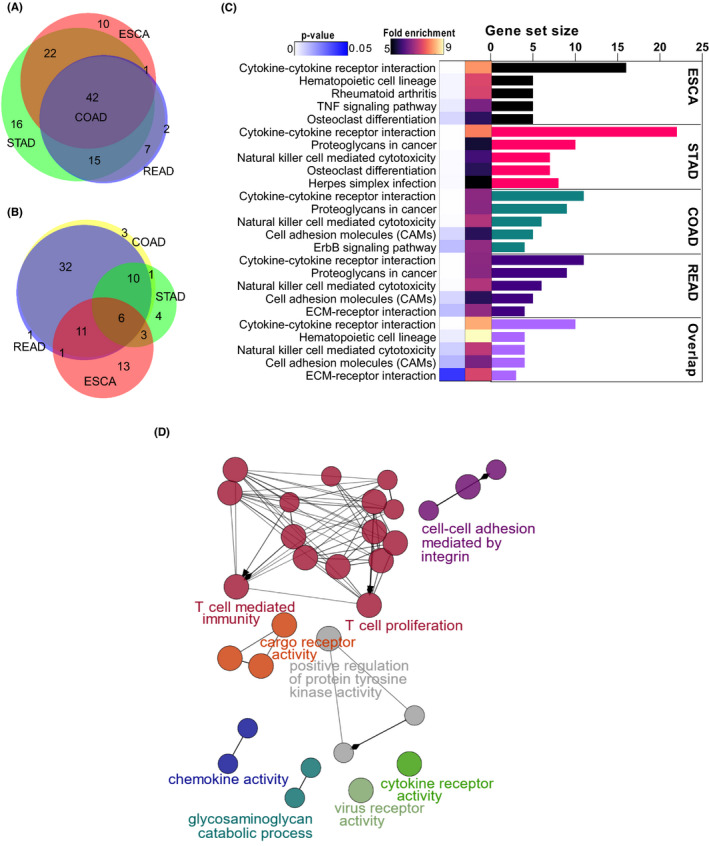
Enrichment and pathway analysis of SMPs in gastrointestinal tumours. (A and B) Venn illustration shows the overlap of SMPs isoforms overlap in various GIACs (ESCA, STAD, COAD and READ) oesophageal carcinoma and stomach, colon and rectum adenocarcinomas respectively. (A) represents upregulated genes, whereas (B) represents downregulated genes Venn illustration of GIACs. (C) KEGG enrichment analyses were performed for overlap of GIACs using SMPs isoform data (*p* < 0.05, Benjamin <0.01). The representative top five enrichment terms were displayed. KEGG pathways analyses were performed utilizing 42 overlapping genes identified in (A). We found that 10 genes related to the cytokine–cytokine receptor interaction pathway were consistent in overexpressed gastric tumours. (D) Genes linked with key gene ontology terms are shown in the ClueGO functional network of the 42 upregulated genes. The statistical significance of the enrichment term is shown by the node size, which is based on the computed *p*‐values <0.05

The transcriptome data enabled us to fine‐tune the previous classification of 20,344 putative protein‐coding genes into different categories based on their expression across gastric tumours compared to non‐tumour tissue types. Here, we used a majority decision‐based (MDM) algorithm to predict a complete set of gastric cancer‐associated human secretome and membrane proteome. Tumour and non‐tumour data sets were analysed for which genomic, epigenomics and clinical details were publicly available (Figure 1A). Differentially expressed protein‐coding genes from GIACs were sorted into three groups, including (1) secretory and membrane protein isoforms (Figure 1B), (2) secretory and (3) membrane (Figure S1A,B).

A functional gene ontology (GO) analysis was performed for each subgroup of differentially expressed tumours (as identified in Figure 1B). Protein‐coding genes with one secretory and one membrane isoform were significantly enriched in ‘cytokine–cytokine receptor interaction’ (CCRI) which was consistent in all four analysed gastric tumours (Figure 2C) as well as in the upregulated overlap between groups. Upregulated and downregulated genes, with multiple splice variants have been classified in Figure 2A,B respectively, based on the presence of membrane‐spanning and secreted protein isoforms.

Analysis of overlap SMPs offered insight into the upregulated biological functions in GIACs. We conducted a Cluego analysis to delineate the biological roles our data that demonstrated these genes were significantly associated with T‐cell‐mediated immunity and cytokine receptor activity (Figure 2D).

**FIGURE 3:**
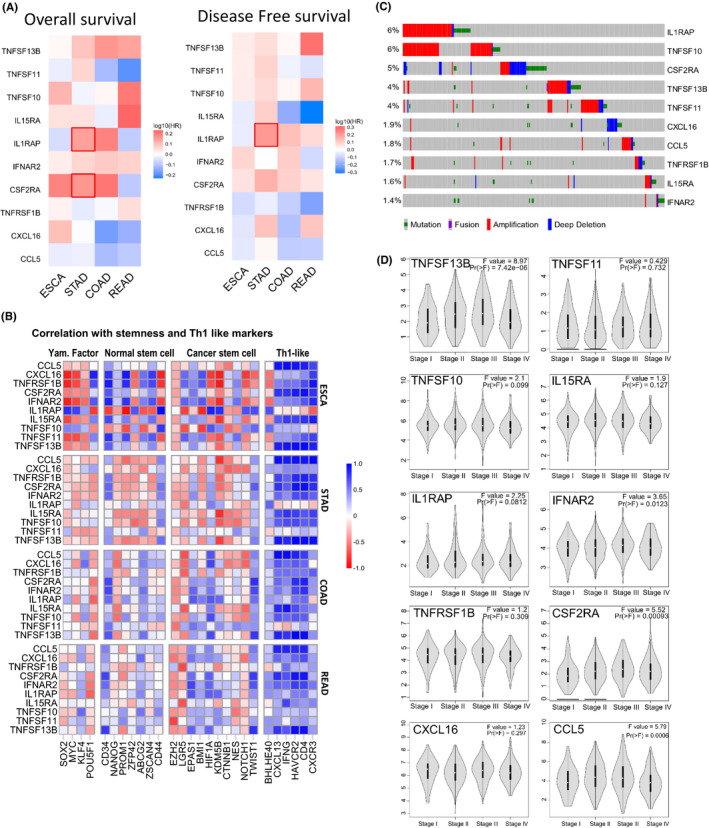
The prognostic power of the cytokine–cytokine‐associated genes signature. (A) Survival analyses of each gene in gastric tumours were performed using the Mantel–Cox test and *p*‐values are shown. (B) Correlation between cytokine signature and stemness markers. Person's correlation coefficient, two‐tailed *p*‐value, 95% confidence. (C) Gene mutations and copy number alteration of 10 cytokines core genes. Each row represents a gene and each column represents a sample. Genes are ranked from high to low somatic alteration frequency. IL1RAP and TNASF10 show the highest somatic mutation frequency among these samples. (D) CCRI expression is stratified by the clinical stage in GIACs. The log2(TPM+1) differential gene expression data is displayed on the *y*‐axis

The upregulated values of CCRI in stomach adenocarcinomas were also evident in the clinical data analyses. After adjusting for clinical factors, an adverse relationship between IL1RAP and survival was detected, which was significant for both overall survival and disease‐free survival (Figure 3A).

Using the Kyoto Encyclopaedia of Genes and Genomes (KEGG), we identified 10 overlapping genes (CCL5, CXCL16, TNFRSF1B, CSF2RA, IFNAR2, IL1RAP, IL15RA, TNFSF10, TNFSF11 and TNFSF13B) that function primarily via cytokine–cytokine receptor interaction. We calculated the somatic copy number alteration (SCNA) and mutation frequency in the gastric‐cancer cohort of 1216 patients by focusing on these genes (Figure 3C). The overall frequency of DNA aberration was low, varying between 1% and 6%. IL1RAP and TNFSF10 had the highest amplification frequency, followed by CSF2RA, TNFSF13B and TNFSF11. CSF2RA had the highest number of deep deletions consistent with their tumour promoter role in cancer development (Figure 3C).

We also computed the correlation of 10 CCRI‐related genes against mRNA expression of published stemness markers, which revealed significant correlations for most tumours. IL1RAP was positively correlated with other core stem cell factors: CD34, H1F1A and TWST1 (Figure 3B). CCRI was examined in GIACs and found to be increased in each successive stage of cancer, increasing expression levels from stage I to stage III, then whereas, decreased in stage IV (Figure 3D).

CCRI expression was used to interrogate the new neoplasm event type in the TCGA data set comprising the expression profile of metastatic samples. We compared metastatic samples to primary TCGA samples, metastatic CCRI levels were found to be significantly higher in most cases (Figure S2).

**FIGURE 4:**
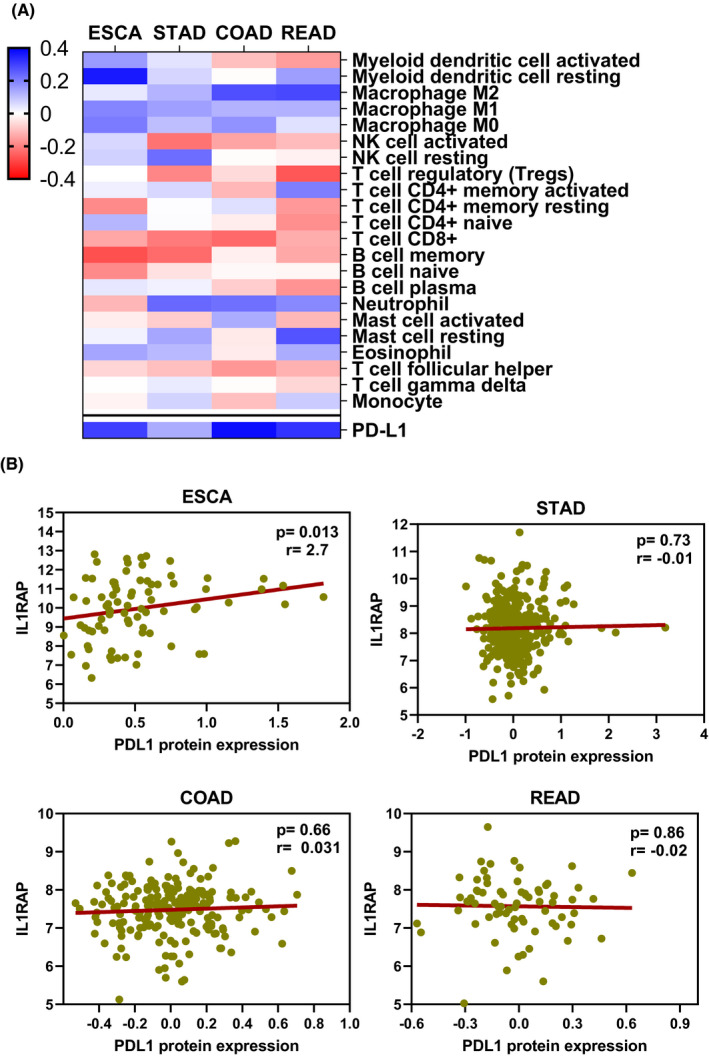
Association of SMPs with Immune microenvironment. (A) Heatmap showing the correlation of significant tumour‐infiltrating immune cell abundance with IL1RAP scores utilizing CYBERSORT (spearman's *p* < 0.05, |correlation| >0.3). Red depicts negative correlations, and blue depicts positive correlations. The correlation with PD‐L1 mRNA expression is shown as a point of comparison. (B) IL1RAP and PD‐L1 protein expression plotted against Spearman correlation, as calculated from ESCA, STAD, COAD and READ

We calculated associations between IL1RAP and individual types of immune cells to determine whether other potential interactions were occurring between IL1RAP and the tumour microenvironment. We used CIBERSORT^40^ to score 22 immune cell types in TCGA tumour samples based on their relative abundance.^41^ These cell types included macrophages, natural killer (NK) cells, dendritic cells and mast cells, monocytes, eosinophils and neutrophils. We measured the association between IL1RAP and the average fraction of individual immune cell types for each TCGA gastric tumour. We also assessed the difference between active and resting fractions of NK cells, CD4+ T cells and macrophages to identify the functional activation of various immune cells (Figure 4A). A recent study demonstrated that immunotherapy is triggered by the activation of peripheral CD4+ T cells and subsequent killing of tumour cells.^42^ The gastric tumours had a negative correlation between IL1RAP and the fraction of T‐cell populations and B‐cell populations. These data are in line with our finding that PD‐L1 protein expression is lower in these tumours (Figure 4B), implying that immune checkpoint blockade is unsuccessful and an alternative immune evasion mechanism may be present.

In GIACs there was a significantly negative association between IL1RAP and the probability of overall survival (OS) or disease‐free survival (DFS) (Figure S3A,B). IL1RAP expression is significantly increased in each successive stage of stomach adenocarcinomas compared with adjacent non‐malignant tissue (Figure S3C,D). We further optimized the IL1RAP siRNAs in vitro utilizing gastric cancer cells lines, enabling its use for further experiments (Figure S3E).

**FIGURE 5:**
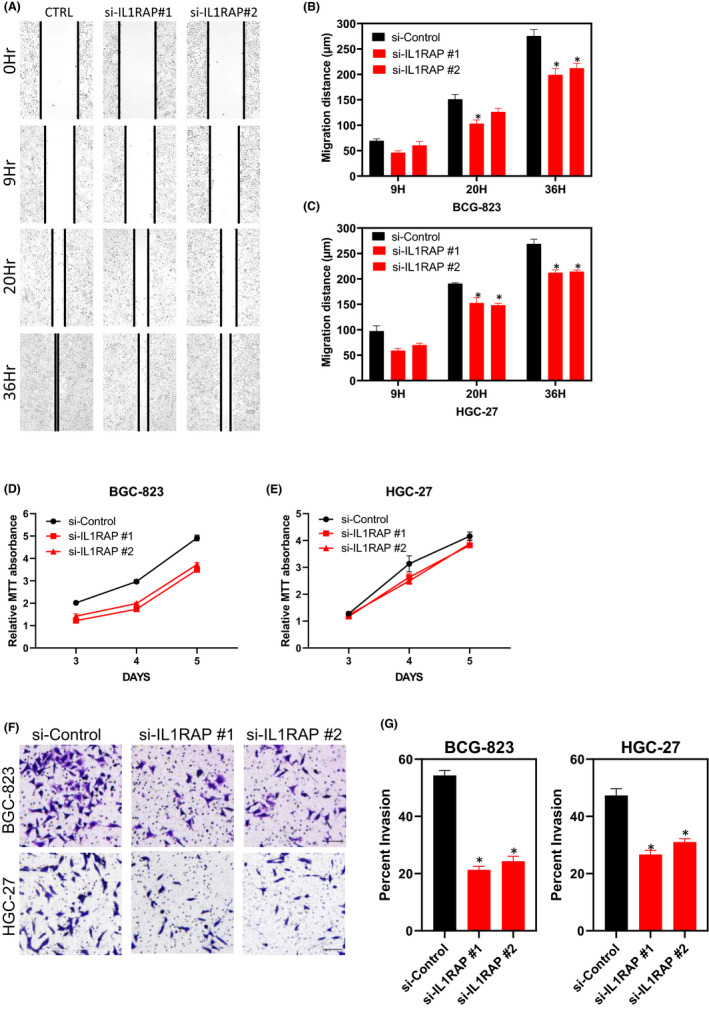
IL1RAP reduces the proliferation and migration capacity of GIACs. (A, B and C) In vitro scratch assays revealed that silencing IL1RAP significantly decreased stomach cancer cell migration (*n* = 3). Data are mean ± SEM; *p < 0.05. (D and E) Stomach cancer cells proliferation was quantified by MTT assay (*n* = 3). (F and G) Matrigel invasion assay was used to evaluate cell invasion capability. Data are mean ± SEM of three independent experiments (*p < 0.05)

We first studied the effects of IL1RAP silencing on migration and invasion of gastric derived cancer cells. In wound healing and Matrigel invasion experiments, silencing IL1RAP severely decreased cellular migration and invasion when compared to controls (Figure 5A–C and F,G).

We then examined whether gastric cancer cells rely on IL1RAP expression levels for proliferation, to indicate if IL1RAP has a functional role in cancer cell progression. The siRNAs‐mediated knockdown of IL1RAP resulted in a substantial reduction in gastric cancer cell proliferation (Figure 5D,E).

### 3.2 Transcriptional signatures of IL1RAP silencing

**FIGURE 6:**
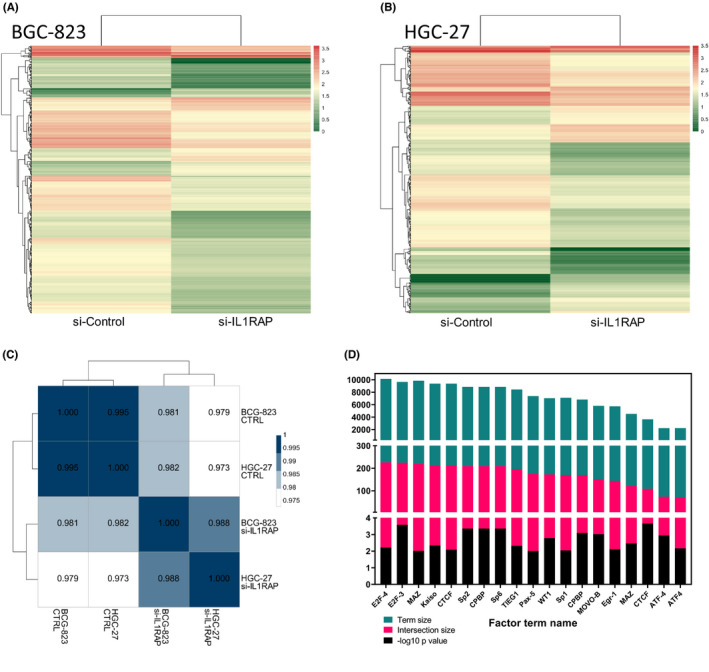
RNA sequencing of BCG‐823 and HGC‐27 cell line. (A and B) Heatmap of differentially expressed genes in BCG‐823 and HGC‐27 stomach cancer cells. Fold difference (|log2(Fold Change) |>1) and the significance level (*q*‐value <0.05). The colour represents log10 (expressed level +1). (C) The Pearson correlation (coefficient; *r*) analysis between samples is based on the expression levels of all genes or transcripts obtained by sequencing. (D) Identification of regulatory motif from DEGs in BCG‐823 cells

To investigate the potential clinical use of the IL1RAP expression profile, we analysed the transcriptomes of two stomach cancer cell lines (BCG‐823 and HGC‐27). We transiently knocked down IL1RAP, using siRNA‐mediated transfection. We first identified the differentially expressed genes (DEGs) in each cell line compared with controls (Figure 6A,B). We found a similar group of genes differentially expressed in both stomach cancer cell lines, indicating a presumptive functional role of IL1RAP in cancer progression. To further confirm these results, correlation analyses were performed between four samples using genetic expression. We found that both cell lines exhibited a positive correlation within the control (BCG‐823 control and HGC‐27 control) or si‐IL1RAP (BCG‐823si‐IL1RAP and HGC‐27si‐IL1RAP) groups (Figure 6C). These results indicate that IL1RAP knockdown led to a consistent inhibition or activation of a similar group of genes between cell lines. The transcription factor family E2F is divided into transcriptional activators (E2F1 to E2F3A) and repressors (E2F3B to E2F8). Members of the E2F family have been established as cell cycle regulators and mediators of proliferation and apoptosis and recently implicated in human cancer.^43^ We similarly found a significant enrichment in E2F‐4 and E2F‐3 transcription factors with the enrichment analysis of differentially expressed genes in carcinoma (Figure 6D).

### 3.3 Identification of potential immune subtypes of GIACs

**FIGURE 7:**
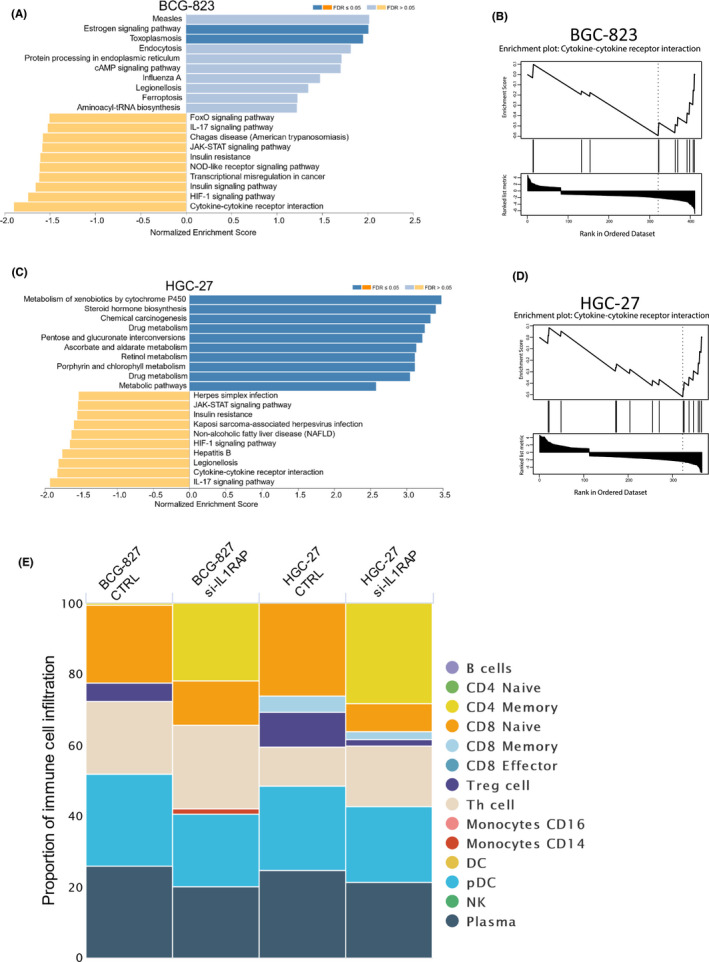
Identification of potential regulatory pathway. (A and C) Enrichment analysis of DEGs regulating the immune‐related genes in BCG‐823 and HGC‐27 stomach cancer cells. The bar chart shows normalized enrichment score (NES) in GSEA with direction and the false discovery rate (FDR) for the categories is ≤0.05. (B and D) GSEA enrichment plot showing the rank distribution and enrichment score of CCRI. (E) RNA seq results were used to estimate the relative proportion of tumour‐infiltrating immune cells

A total of 446 (BCG‐823) and 390 (HGC‐27) genes were differentially expressed after IL1RAP knockdown. Subsequently, GSEA enticement analysis suggested that five signalling pathways (e.g. cytokine–cytokine receptor interaction, IL‐17, JAK‐STAT, HIF‐1 and Insulin resistance) were significantly inhibited in both cell lines after silencing IL1RAP (Figure 7A,C). We did not find a consistent pattern of activated pathways. Since the inhibition of cytokine–cytokine receptor pathway may specifically associate with silencing of IL1RAP, in stomach cancer cells, the enrichment of CCRI associated genes was used to construct enrichment plots (Figure 7B,D).

The CIBERSORT‐mediated deconvolution algorithm was utilized to identify tumour‐infiltrating immune cells from RNA seq results. Both gastric cancer lines showed a similar pattern of immune infiltration. We observed that IL1RAP knockdown cells had substantial infiltration of T‐helper cells and CD4 memory cells but lower infiltration of Tregs and CD8 naive cells compared with control cells (Figure 7E).

Long non‐coding RNAs (lncRNAs) are crucial biological regulators and their role in cancer biology is emerging as the understanding of their upstream and downstream target genes is increasing.^44^ A total of 40 and 31 ncRNA‐related genes were differentially expressed in BCG—823 and HGC‐27 cell lines respectively (Figure S4A,B). Twenty‐two ncRNAs were differentially expressed in both cell lines. We identified ncRNA based on transcription factors regulated upstream for each DEGs cohort. We found that MYC‐related targets, which have aberrant expression in up to 70% of human cancers,^45^ were significantly expressed in both cohorts (Figure S4C,D).

## 4 DISCUSSION

The cancer secretome and membrane proteome represent a potential source of gastric tumour biomarkers. The extracellular presence of these proteins provides access to physiological targets via biofluids.^5^, ^6^, ^14^ In addition to acting as a reservoir of biomarker candidates, the cancer secretome and membrane proteome are recognized to have an important role in tumour oncogenesis, invasion and metastasis.^17^, ^18^, ^19^, ^20^ In this study, we aimed to evaluate the functional difference of encoded proteins in relation to gastric cancer secretome and membrane proteome. We analysed 957 adenocarcinomas of the stomach, oesophagus, colon and rectum to examine the functional association of SMPs with gastrointestinal tract adenocarcinomas (GIACs).^46^, ^47^, ^48^, ^49^ The results reported herein illustrate that a small group of secretory and membrane isoforms were noticeably enriched in cytokine–cytokine interaction pathway and are believed to be a source of oncogenesis. Particularly, cytokine–cytokine receptor interaction pathway encoded genes showed significantly higher levels of expression in GIACs compared with non‐tumour tissue. Cytokines are secreted or membrane‐bound signalling molecules. Upregulation of cytokine–cytokine receptor interaction pathway is suggested to promote immunosuppression, angiogenesis and therapeutic resistance in gastrointestinal adenocarcinomas.^50^ Immune and non‐immune cell interactions are mediated by cytokines in the tumour microenvironment (TME). A recent publication explained how the TME enables malignant cells to co‐evolve with immune responses in lung adenocarcinoma.^51^ Several cytokines (among interleukins) are especially important in tumour development and progression. The pleiotropic activity of interleukins in cancer is defined by a plethora of cellular sources, receptors, signalling pathways and even dose dependence. Overall, interleukin activity can be cell‐specific and includes cancer initiation, tumour growth and tumour control.^52^

IL1RAP is a promising cell‐surface marker and has been found to be significantly upregulated in gastric adenocarcinomas compared with normal adjacent tissue. IL1RAP is a co‐receptor for IL‐1 and it is known to transduce in IL‐1 signalling, which results in cell survival and proinflammatory gene expression.^53^ Here, we show that disruption of IL1RAP expression through RNA interference significantly impedes stomach pathogenesis without perturbing healthy haematopoiesis in the absence of immune effector cells.

At first, the finding of an increased CCRI expression profile from stage I to stage III, with decreasing expression in stage IV in most cases, seemed perplexing. As previously described,^54^ this decrease in expression in the final stage might be attributed to the gradual loss of tumour memory from its tissue of origin and transition to a stem cell‐like state.

The TME is important for diagnosis and treatment response.^55^ Interleukins identified in this study were upregulated in GIACs and may be involved in stimulating tumour cell growth. This stimulation may involve signalling specific gut hormones in GIACs.^55^ Immunosuppressive cytokines and chemokines have been shown to affect T‐cell growth, migration, and function in the tumour microenvironment.^56^ CD4+ T cells have long been thought to be tumour suppressors, CD4+ T cells have an intrinsic capacity to stimulate cytotoxic T lymphocytes (CTLs), and Th1 effector cells have been demonstrated to suppress tumour development and promote CTL function.^56^, ^57^ Recent studies suggest that chronic alcohol consumption and Helicobacter pylori infection may promote gastric tumorigenesis through IL‐10 suppression and reduced CD8+ cell infiltration.^58^, ^59^ Many cancers have increased expression of secretory pathway machinery.^60^ Our data suggest a consistent pattern of CCRI in which cancer cells alter their secretome profile by decreasing tissue‐specific component synthesis and increasing tumorigenic factor production. Among these factors, elements like IL1RAP may be inhibitory in the activation of T cells and may be a potential target for immunotherapy.^42^ IL1RAP is one of the most commonly mutated secretory and membrane protein isoforms in GIACs. In line with this hypothesis, CCRI has the potential to be used as a therapeutic target. In all presently investigated tumours, IL1RAP expression was significantly increased with disease progression. In ESCA, STAD, COAD and READ high expression was associated with a poor overall survival rate.

In conclusion, the gastric cancer secretome and membrane proteome functional heterogeneity and direct interaction in a number of tumorigenic and metastatic pathways emphasize the significance of this group of proteins in tumour pathophysiology. These findings provide a compelling argument for targeting this group in anti‐cancer therapeutic applications. Furthermore, the CCRI biomarker candidates for gastric tumours, reported herein, particularly IL1RAP, are likely to help in the development of less invasive and more accurate diagnostic and potentially therapeutic modality.

## CONFLICT OF INTEREST

The authors declare no conflict of interest.

## AUTHOR CONTRIBUTIONS

**Adeel ur Rehman contributed to** conceptualization (lead); formal analysis (lead); visualization (equal); writing—original draft (lead); and writing—review and editing (equal). **Per Olof Olsson contributed to** validation (equal); visualization (equal); and writing—review and editing (equal). **Aleena Akhtar contributed to** formal analysis (equal); and writing—review and editing (equal). **Arshad Ahmed Padhiar contributed to** validation (equal) and visualization (equal). **Hanyang Liu** contributed to validation (equal) and visualization (equal). **Yi Dai contributed to** resources (equal). **Yu Gong contributed to** investigation (equal). **Yan Zhou contributed to** iInvestigation (equal). **Naveed Khan contributed to** formal analysis (equal). **Haojun Yang contributed to** resources (equal). **Liming Tang contributed to** funding acquisition (lead) and supervision (lead).

## Supporting information

Figure S1‐S4

## Data Availability

The data that support the findings of this study are available from the corresponding author upon reasonable request.
