# Supplementary figures and images for: Systematic molecular analysis of the human secretome and membrane proteome in gastrointestinal adenocarcinomas
Source: J Cell Mol Med. 2022 Apr 29;26(12):3329–42. doi: 10.1111/jcmm.17338 (PMC9189341; doi:10.1111/jcmm.17338)

# Supplementary Figure 1

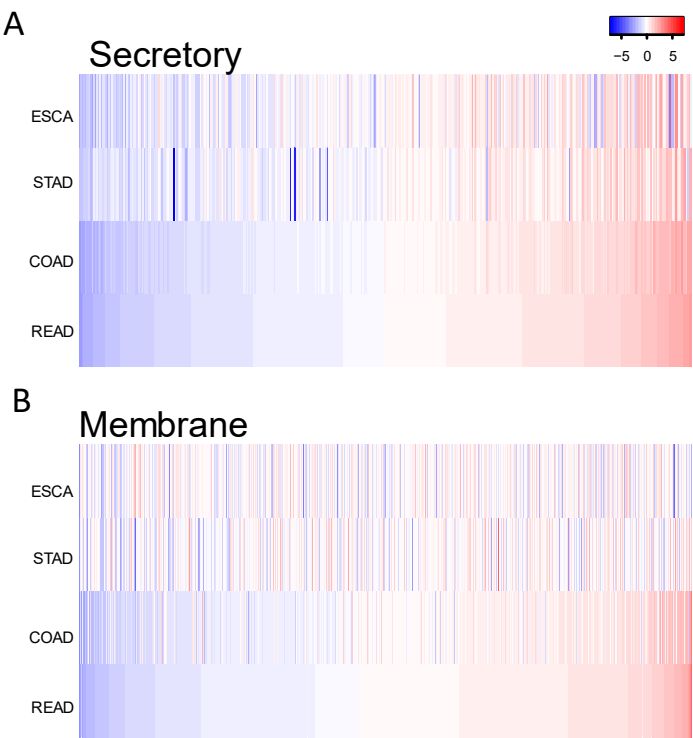

# Supplementary Figure 2

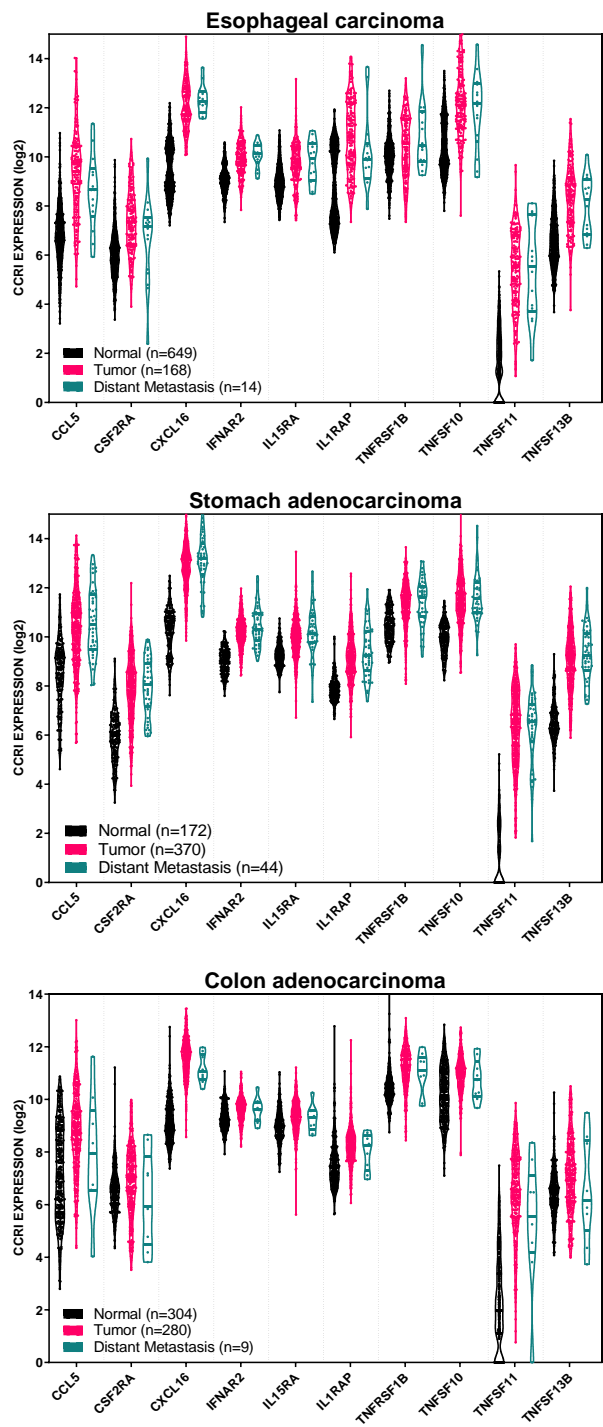

# Supplementary Figure 3

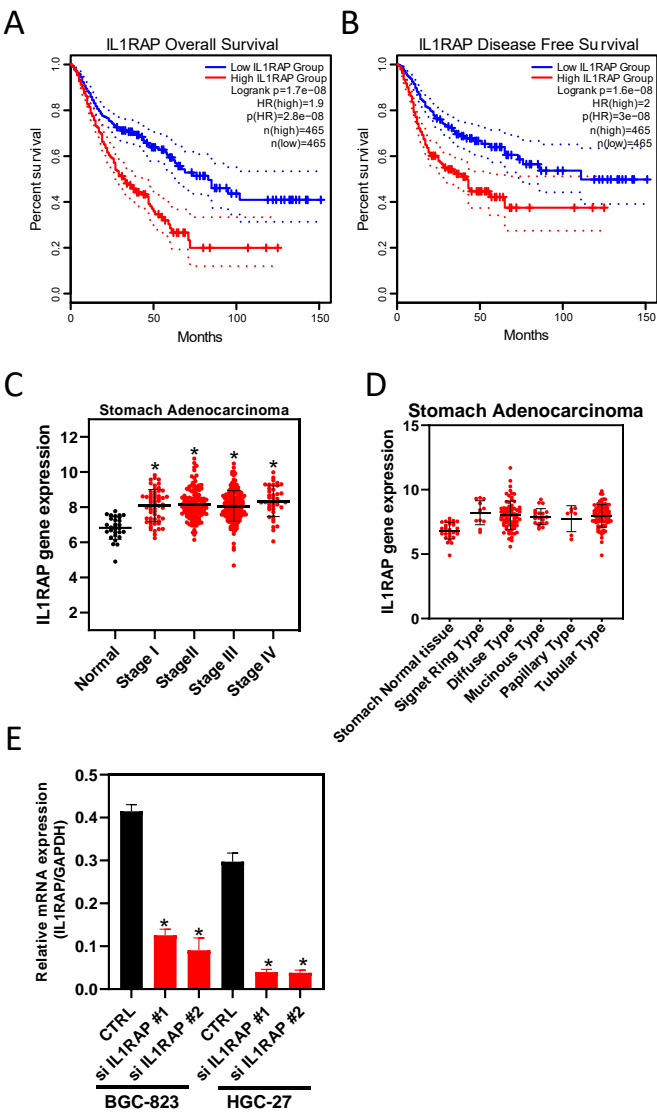

# Supplementary Figure 4

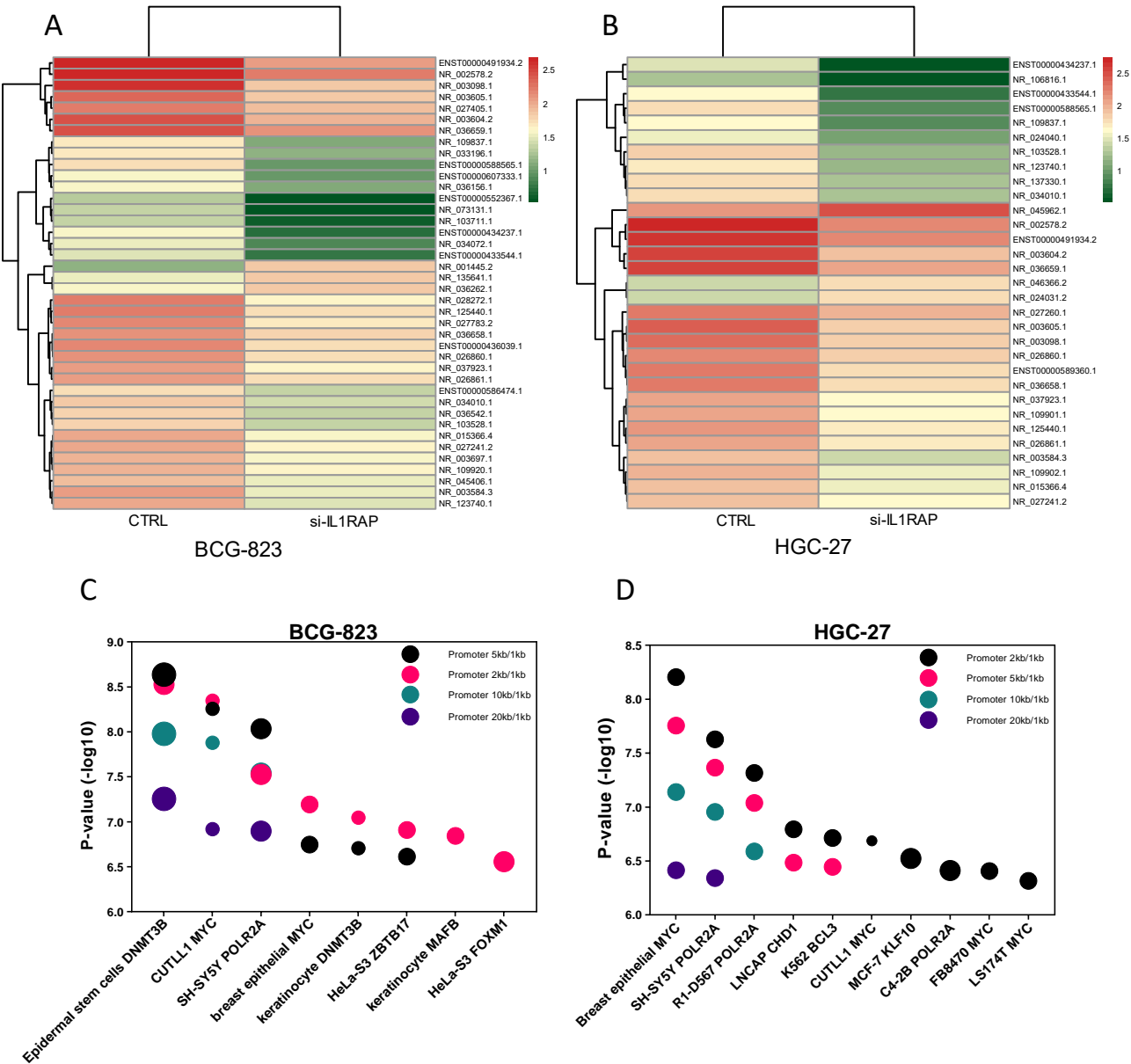

Supplement: Supplementary file 1 — Figure S1‐S4 [file JCMM-26-3329-s001.pdf]
